# Chemistry of Mezcal: Volatile Profile of Artisanal Mezcal Made from Wild Agaves of Oaxaca

**DOI:** 10.3390/foods14071222

**Published:** 2025-03-31

**Authors:** Rosa Elvira Sánchez-Fernández, Artemio Pérez-López, Anabel Morales-Solis, Yesenia Manilla-Tellez, Erika Daniela Reyes-Carmona, Graciela Avila-Uribe

**Affiliations:** 1 Laboratorio Nacional de Investigación y Servicio Agroalimentario y Forestal (LANISAF), Universidad Autónoma Chapingo, km 38.5, Carretera Mexico-Texcoco, Texcoco 56230, Mexico; 2 Posgrado en Ciencia y Tecnología Agroalimentaria, Departamento de Ingeniería Agroindustrial, Universidad Autónoma Chapingo, km 38.5, Carretera Mexico-Texcoco, Texcoco 56230, Mexico; moralessolis.anabel@gmail.com (A.M.-S.); yes.matez456@gmail.com (Y.M.-T.); danireyesc@gmail.com (E.D.R.-C.); 3 Estancia Posdoctoral Por Mexico, Secretaría de Ciencia, Humanidades, Tecnología e Innovación, Posgrado en Ciencia y Tecnología Agroalimentaria, Universidad Autónoma Chapingo, km 38.5, Carretera Mexico-Texcoco, Texcoco 56230, Mexico; avilauribeg@gmail.com

**Keywords:** volatile compounds, GC-MS, mezcal, HS-SPME, agave

## Abstract

Mezcal is a distilled beverage with a complex chemical profile defined by volatile organic compounds and physicochemical properties that determine its sensory attrib-utes. This study analyzed nine artisanal mezcals produced from four wild agave species in Oaxaca using solid-phase microextraction (SPME) and gas chromatography–mass spectrometry (GC-MS) to identify key volatile compounds for traceability and quality control. A total of 82 volatile compounds were identified, with esters, terpenes, and higher alcohols being the most abundant. Eight key compounds, including ethyl acetate, acetic acid, 1-butanol, furfural, methanol, and 2-methyl-1-propanol, were quantified due to their significant impact on mezcal’s quality and authenticity. Additionally, 1,2,3-trimethyl-benzene, nerolidol, and terpinolene were identified as exclusive compounds for differentiating mezcal by agave species and storage duration. The findings highlight the influence of fermentation, distillation, and storage conditions on mezcal’s chemical profile and demonstrate the importance of standardized analytical methods for product authentication. Proper management of variables during fermentation and optimization of the final distillation cuts is necessary to fully comply with regulatory parameters and ensure product quality. By establishing a catalog of compounds that characterize the mezcals, this study provides a scientific basis for improving quality control, ensuring regulatory compliance, and enhancing the traceability of mezcal in high-value markets. The next step is to validate the key volatile compounds with a larger sample and evaluate their reproducibility under different production and storage conditions.

## 1. Introduction

In recent years, mezcal has gained widespread acceptance among consumers in numerous countries, establishing itself as an emblematic beverage with a diverse organoleptic profile. Its characteristics—appearance, flavor, aroma, and mouthfeel—are directly linked to the agave species used and the production process employed. Despite its growing popularity [1], there is still a significant gap in scientific knowledge regarding the specific volatile composition of mezcal made from wild agave species and how production variables influence its chemical profile. Given this context, the present study aims to address the following research question: How do the species of wild agave, production methods, and storage conditions influence the volatile profile and physicochemical pro- perties of artisanal mezcal?

The specific diversity of agave, shaped by its genetic expression, interacts in a complex manner with climatic, edaphic, and altitudinal factors, resulting in a unique chemical profile for each plant. This genetic and environmental specificity is directly reflected in the sensory properties of the distilled spirit, including its aroma, flavor, and texture, which arise from biochemical synthesis influenced by specific secondary metabolites. This phenomenon underscores the importance of the genotype–environment interaction in defining the organoleptic characteristics and overall quality of the final product. Furthermore, the production process—encompassing key stages such as harvesting, cooking, milling, fermentation, distillation, and bottling—plays a crucial role in developing volatile organic compounds (VOCs), texture, and sensory profile. This highlights the significance of the interaction between the production process’s technical aspects and the distilled spirit’s perceived quality [2]. Finally, the type of packaging, storage material, and aging time also play a significant role in determining the final composition of VOCs in the product [3,4]. Together, these differences reflect not only traditional practices but also regional adaptations that enhance the product’s identity. This diversity, resulting from the interaction between genetics, environment, and technique, positions mezcal as a valuable model for studying fermentation and distillation processes within the context of traditional agro-industrial systems [5].

Among the most commonly used species in the Sierra Sur region of Oaxaca, *Agave angustifolia* Haw stands out for its high production volume, adaptability to diverse climatic conditions, high-quality sugars, and relatively short cultivation cycle. *Agave potatorum*, a wild species with slow maturation, is valued for producing mezcal with a refined aromatic profile, characterized by sweet and fruity notes with a delicate smoky touch [5,6]. *Agave marmorata* Roezl, an endangered species, grows in hills and deciduous lowland forests, often in challenging natural environments, and yields a mezcal with a complex aromatic profile featuring herbal, floral, fruity, and spicy notes [6]. Lastly, *Agave karwinskii* Zucc., known as madrecuishe, barril, or cuishe, is distinguished by its resilience, easily adapting to poor and dry soils. This agave produces a high-quality mezcal, appreciated for its elegant flavors and aromas with herbal, earthy, and mineral notes [6,7]. This sensory richness in mezcal highlights the importance of understanding and optimizing the factors that influence its aromatic composition, not only to enhance the value of agave biodiversity but also to strengthen the authenticity and competitiveness of mezcal in global markets [5,7].

The aroma and flavor of distilled alcoholic beverages result from a complex mixture of numerous VOCs and non-VOCs, which collectively define their sensory attributes, texture, and consumer acceptance [8]. The most notable VOCs that contribute to the aroma and body of mezcal include esters, aldehydes, ketones, organic acids, furans, and terpenes [3,9,10,11]. Table 1 shows the properties and origin of some metabolites quantified in this work. Previous studies on other distilled spirits, such as tequila (*Agave tequilana* Weber), have analyzed VOCs using various techniques, including ultraviolet–visible (UV–Vis), absorption spectroscopy [12], Raman spectroscopy [13], multivariate analysis of Fourier Transform Infrared (FTIR) Spectroscopy [14], and gas chromatography [15,16,17]. These methods enable the identification and analysis of a wide variety of volatile substances, providing essential information about their components, which aids in characterization and authentication. In the specific case of mezcal produced from *A. potatorum*, *A. Angustifolia* Haw, *A. Marmorata* Roezl, and *A. Karwinskii* Zucc., there remains a significant gap in scientific knowledge regarding its chemical composition and specific characteristics. This is particularly relevant given that these species play a fundamental role in mezcal production within a Denomination of Origin (DO) region, especially in the mezcal-producing area of Miahuatlán de Porfirio Díaz, Oaxaca [1]. The lack of detailed studies on the composition and properties of these distilled spirits not only limits the valorization of the product but also hinders its differentiation in a competitive global market. In such a market, recognizing the sensory, chemical, and cultural characteristics of products with DO is essential for their positioning and commercial success [2].

Currently, the use of gas chromatography–mass spectrometry (GC-MS) is considered optimal for the detection of VOCs in mezcal and different organic materials [6,26,27,28,29]. This highly accurate analytical method allows for the separation, identification, and quantification of different VOCs in a sample. The techniques used for the extraction of components in alcoholic beverages include component concentration via microdistillation, liquid–liquid extraction, and solid-phase microextraction (SPME). Among these, headspace (HS) SPME is currently the recommended sampling technique for the analysis of alcoholic beverages because it does not require solvents and minimizes sample preparation [5,6,11,18,30].

In this context, the present study aims to analyze the physicochemical properties and volatile compounds in artisanal mezcal produced from the most economically significant agave species in the Sierra Sur region of Oaxaca. Using GC-MS and statistical tools, the study seeks to identify key volatile compounds and establish a catalog of chemical compounds that characterize the samples to ensure authenticity and enhance the traceability of production processes, thereby guaranteeing the quality of mezcal in competitive and high-value markets.

## 2. Materials and Methods

### 2.1. Reagents

The reagents used for the identification and quantification of the main volatile compounds were purchased from Sigma-Aldrich^®^ (St. Louis, MO, USA) and J.T. Baker^®^ (Radnor, PA, USA), ensuring high standards of purity and quality. From Sigma-Aldrich^®^, the following compounds and their respective purities were used: acetic acid (100.0%), furfural (99.2%), 1-propanol (100.0%), 2-methyl-1-propanol (99.9%), diethyl acetal aldehyde (99.4%), 1-butanol (100.0%), methanol (99.98%), ethyl acetate (99.9%), 3-methyl-1-butanol acetate (100.0%), 2-methyl-1-butanol (99.5%), 3-methyl-1-butanol (99.6%), propionic acid (99.5%), 1-hexanol (98.0%), linalool (97.0%), methyl salicylate (99.4%), and formic acid (95.0%). Additionally, absolute ethanol (99.5% purity) from J.T. Baker^®^ was used.

### 2.2. Mezcal Samples

The mezcal samples analyzed correspond to different species of agave cultivated in wild environments, including *Agave potatorum* (mezcal tobalá), *Agave karwinskii* Zucc. (mezcals bicuishe, madrecuishe, and tobasiche), *Agave angustifolia* Haw. (mezcal espadín), and *Agave marmorata* Roezl. (mezcal tepeztate). These agaves were harvested within the territorial limits of the community of Sitio Xitlapehua (16°21′45″ N, 96°32′30″ W) and San Luis Amatlán (16°23′00″ N, 96°30′00″ W), Oaxaca, Mexico. The predominant climate in the region is semi-warm subhumid with summer rainfall. The mezcal production process was carried out in two artisanal factories called “palenques” following an artisanal scheme that includes the following stages: the harvest of the agave stem was performed manually using machetes; the cooking was carried out in underground pit ovens, using firewood as heat source, with an exposure time of 72 h; the chopping of the cooked agave stem was performed manually with machetes and their milling with a wooden mallet; the fermentation occurred spontaneously using the natural microflora present in the environment; subsequently, the distillation was carried out in copper stills, and finally, the bottling was carried out in glass bottles. The mezcal sampling was carried out in 250 mL Pyrex glass jars with an airtight seal, and the analyses of physicochemical variables and volatile compounds were conducted at the Laboratorio Nacional de Investigación y Servicio Agroalimentario y Forestal (LANISAF) at the Universidad Autónoma Chapingo.

### 2.3. Physical Properties of the Samples

#### 2.3.1. Viscosity

The flow ramp tests were conducted using a Discovery Hybrid HR-3 rheometer ((TA Instruments, New Castle, DE, USA) equipped with a concentric cylinder geometry (inner cylinder: 42 mm × 28 mm; outer cylinder: 78.5 mm × 30.15 mm), with a gap of 5920 µm. The samples were thermostated at 25 ± 0.1 °C using a Peltier temperature control system. The experimental protocol involved applying a shear rate ramp within a range of 0.1 to 300 s^−1^, with logarithmic increments and a stabilization time of 5 s at each measurement point. Measurements were performed under a shear rate control regime (flow ramp). The viscosity of the samples was measured in triplicate, and the results were expressed in mPa∙s [31].

#### 2.3.2. Density and Alcohol Content by Volume

In the context of mezcal, density and alcohol content are key parameters to ensure the quality and authenticity of the product. The standard NOM-070-SCFI-2016 [32] establishes that the alcohol content in mezcal must range between 35% and 55% vol. Density, in turn, is used as an indirect measure to verify this range, due to the inverse correlation between these two variables. Various factors can influence the density of mezcal, including the type of agave, the presence of non-volatile compounds such as residual sugars, as well as substances derived from wood in aged beverages, and temperature [31].

The density of the samples was determined using a portable densimeter [31] SNAP 51 (Anton Paar, Graz, Austria), which measures density based on the oscillating U-tube principle. The vibratory tube is filled with approximately 2 mL of the sample, and the resulting oscillation frequency, which varies with the mass of the liquid, is used to calculate the density. The device includes an integrated temperature sensor that automatically corrects the measured values to 20 °C. Each sample was analyzed in triplicate to ensure precision and reproducibility, and the results were reported as the average of the measurements in g/cm^3^.

The alcohol content by volume (% *v*/*v*) of the samples was determined using a portable densimeter [33] SNAP 51 (Anton Paar, Graz, Austria), which operates based on the oscillating U-tube principle and correlates the sample’s density with its alcohol content using internal calibration tables. The device automatically adjusted the measured density to 20 °C through temperature compensation. Each sample was analyzed in triplicate, and the results were reported as the average of the measurements.

#### 2.3.3. Total Soluble Solids

Total soluble solids were measured using a portable digital refractometer model HI96801 (HANNA^®^, Hannapro S.A. de C.V., Woonsocket, RI, USA), which has a measurement range of 0–85%. The samples were measured at room temperature, in triplicate, and the results were reported as percentage values.

### 2.4. Determination of Volatile Organic Compounds by HS-SPME and GC-MS

The identification of VOCs was performed using HS-SPME followed by GC-MS, using a fiber of Divinylbenzene/Carbon wide range/Polydimethylsiloxane (DVB/CWR/PDMS, 1 cm, 50/30 µm, Supelco Bellefonte, PA, USA). For the extraction of the volatiles, 5 mL of mezcal was placed in vials for SPME with a capacity of 22 mL. The sample was placed under magnetic stirring at 100 rpm at 25 °C, the SPME fiber was inserted and exposed in the headspace of the vial for 60 min. The fiber was conditioned for 15 min at 180 °C before each injection.

Next, the fiber was removed and introduced into the injector of the gas chromatograph (GC) Agilent 7890B (Agilent Technologies, Inc., Santa Clara, CA, USA) coupled to a mass selective detector (MSD) Agilent 5977A (Agilent Technologies, Inc., Santa Clara, CA, USA) at 180 °C in splitless mode with a desorption time of 15 min. The volatile compounds were separated in a DB-WAX Ultra Inert capillary column (60 m × 250 μm × 0.25 μm) using helium as the carrier gas at a flow rate of 1 mL/min and the following oven temperature program: initial temperature of 40 °C for 4 min, heating up to 100 °C at a rate of 5 °C/min, 200 °C at a rate of 10 °C/min, and a final time of 10 min. Conditions of the mass selective detector were as follows: electron ionization energy of 70 eV, scan range of 30–550 amu, scan speed of 13.8 spectra/s, ionization chamber temperature of 200 °C, and transfer line temperature of 250 °C. The acquisition and processing of the data were performed using MassHunter Workstation software version 6.0 (Agilent Technologies, Inc. 2014, Santa Clara, CA, USA). Each sample was analyzed in quadruplicate, and blanks were used with vials without a sample and vials containing an ethanol–water mixture (60:40) [11,34].

The compounds were tentatively identified by comparing their mass spectra fragmentation patterns with those available in the National Institute of Standards and Technology (NIST) database. Additionally, Kovats retention indices (KRI) were calculated relative to the retention times of a series of alkanes (C_7_–C_40_) [35] using the following equation (Equation (1)):

(1) $$KRI=100\;n+100\;\frac{t_{Rx}-t_{Rn}}{t_{RN}-t_{Rn}}$$

where *n* is the number of carbon atoms in the n-paraffin that elutes before the interest compound (*x*), *t_Rx_*, *t_Rn_*, and *t_RN_* are the retention times of compound *x*, alkane *n*, and alkane *N* (*n* + 1), respectively.

The KRI values were compared with those reported in the NIST database. The relative quantities of each compound were expressed as the percentage of peak area (% area) relative to the total area of each sample and as an average of the four repetitions. The identification of volatile compounds was performed by comparing the mass spectra and Kovats indices with authentic standards (Sigma-Aldrich, ≥95% purity) [36].

### 2.5. Quantification of Main Volatile Organic Compounds

The quantification of the main VOCs identified in mezcal (ethyl acetate, methanol, diethyl acetal aldehyde, 1-propanol, 2-methyl-1-propanol, 1-butanol, acetic acid, and furfural) was performed by HS-SPME and GC-MS as described above and using the DVB/CWR/PDMS (1 cm, 50/30 µm) fiber. Calibration curves were prepared using an ethanol–water solution (60:40) and standards within a concentration range of 10–1000 µg/mL for each compound [11,34]. Formic acid was used as the internal standard (2.5 μL). The correlation coefficients (R^2^) for all compounds were ≥0.995. Table S1 shows calibration information. The concentration of the compounds was expressed in milligrams per liter (mg/L) and milligrams per 100 mL of anhydrous alcohol, following NOM-070-SCFI-2016 (2016) [32].

### 2.6. Statistical Analysis

The values of the physical properties and quantified volatile compounds were analyzed using a one-way analysis of variance (ANOVA) with a Tukey test (α = 0.05) to identify statistical differences between the types of mezcal. Additionally, a Pearson correlation analysis was conducted, and the results are presented in a heatmap.

The qualitatively identified volatile compounds were analyzed using principal component analysis (PCA) and multiple correspondence analysis (MCA) to visualize the relationships between the compounds and the agave species in a common space. The results are presented as correspondence maps and heatmaps. All statistical analyses were performed using R software (version 4.3.1) [37] and the RStudio integrated development environment (version 2023.06.01) [38].

The PCA axes in the study are directly related to mezcal’s sensory properties be-cause they capture the variance in chemical composition that influences aroma and flavor. The closer a mezcal sample is to a specific VOC on the PCA plot, the more that compound contributes to its sensory profile. Similarly, MCA helps to identify unique chemical volatile compounds that differentiate mezcals based on agave species and production techniques, which in turn affect the sensory experience. ANOVA further supports these findings by highlighting significant differences in chemical composition that can be linked to distinct sensory attributes. Together, these statistical methods provide a robust framework for understanding how the chemical composition of mezcal translates into its complex sensory profile.

## 3. Results and Discussion

### Volatile Compounds and Physical Properties of Mezcals

Recent studies demonstrate that the diversity of volatile compounds, such as alcohols, esters, aldehydes, and organic acids, significantly depends on the agave species used, environmental conditions, production practices, and seasonal variations [6,8,39]. The mean comparison analysis indicated that, except for methanol, diethyl acetal aldehyde, 1-propanol, and 2-methyl-1-propanol, all other evaluated variables showed significant differences (*p* ≤ 0.5) in the analyzed samples (Table 2).

It is worth mentioning that acetic acid, furfural, total soluble solids, alcohol content, ethyl acetate, 1-butanol, viscosity, and density exhibited differences among the analyzed mezcals. Therefore, this significant variability among variables allows for differentiation based on agave species, production year, and production techniques, facilitating the identification of origin and authenticity of the product. These results contribute significantly to the establishment of a platform for the characterization of the distilled spirit in the study region, specifically in the exploitation of wild agave species. Table 3 presents the concentration of the eight VOCs in the complete liquid sample and the normalized concentration relative to pure alcohol content, as the mg/100 mL A.A. concentration is the standard recommended by NOM-070-SCFI-2016 for the standardized measurement of compounds in distilled alcoholic beverages such as mezcal.

It was observed that significant variations in volatile compounds such as ethyl acetate, furfural, and higher alcohols (1-propanol, 2-methyl-1-propanol, and 1-butanol) reflect an interaction between agave characteristics, technological variables (such as agave stem (leafless axis) harvesting, cooking, fermentation, and distillation) and the product’s storage period. For example, *A. potatorum* (mezcal tobalá) exhibited higher levels of ethyl acetate, while *A. karwinskii* Zucc. (mezcal tobasiche) and *A. angustifolia* Haw (mezcal espadín) showed the lowest concentrations, demonstrating a direct influence of agave species, technological variables, and storage period on the aromatic profile [8,40] (Table 2 and Table 3). Similarly, the low concentration of 2-methyl-1-propanol in samples with longer storage periods (tobalá 2025, bicuishe 2015, and madrecuishe 2016) could be attributed to the transformation of this compound into esters. Esters are responsible for imparting sweet, fruity, or floral notes, contributing to a more complex and balanced profile over time, which has already been identified as a key factor in differentiating the chemical profile [8,40]. Additionally, studies using techniques such as surface plasmon resonance (SPR) for detecting volatile compounds show that these profiles are useful for distinguishing between mezcals from different agave species, even under similar production conditions [6]. The 1-butanol concentration was higher in mezcal with longer storage periods. The most likely cause is the selective oxidation of primary alcohols to the corresponding aldehydes, which is one of the most difficult transformations to control because of the marked propensity towards over-oxidation to the respective carboxylic acid [41,42], especially if storage in a semipermeable container is contemplated. Fermentation is also a key factor in the development of the aromatic profile, and in the production of artisanal mezcal, it is spontaneous; this type of fermentation consists of adding water to the cooked and previously macerated agave stem in a tank; this mixture is called must [43]. Fermentation is carried out spontaneously, under the action of the microbiota existing in the environment or in the must itself, this process can last from 5 to 10 days [5]. Fermentation represents a key phase in the artisanal production of mezcal, as numerous aromatic compounds, both volatile and non-volatile, are generated during this process, which influence the final quality of the beverage [44]. These compounds include alcohols, ketones, terpenes, acids, phenols, and aldehydes [5,18].

The alcohol content of tobalá 2015 and madrecuishe 2021 mezcals, with values ranging between 44.8% and 50.3% *v*/*v* (Table 2), complies with the requirements established by the standard [33] and highlights the complexity and authenticity characteristic of artisanal products. The variability in alcohol content among mezcals indicates the influence of factors such as agave species, agroecological conditions, distillation methods, and maturation time. These differences are essential for characterizing and differentiating mezcals, allowing the establishment of unique profiles that reflect local conditions and the expertise of the producers. The analysis of volatile compounds and alcohol content can be used to identify specific chemical compounds that ensure the authenticity of the product [11].

A total of 82 VOCs were identified in the analyzed mezcals (see Supplementary Materials, Table S2). The identified compounds are classified into esters (23 compounds), ethers (8 compounds), alcohols (11 compounds), ketones (2 compounds), aldehydes (2 compounds), terpenes (24 compounds), acids (3 compounds), and benzene derivatives (9 compounds), with esters and terpenes being the most abundant.

Among these, the concentrations of ethyl acetate, acetaldehyde diethyl acetal, methyl alcohol, 1-propanol, 2-methyl-1-propanol, 1-butanol, acetic acid, and furfural were quantified (Table 2 and Table 3) due to their significant influence on organoleptic characteristics, quality, and the safety of the final product, in accordance with NMX-V-005-NORMEX-2013. Additionally, other VOCs were consistently identified across all mezcal samples, although they were not quantified, including: 3-methyl-1-butanol acetate, 4-carene, limonene, 2-methyl-1-butanol, 3-methyl-1-butanol, hexanoic acid ethyl ester, octanoic acid ethyl ester, nonanoic acid ethyl ester, 5-methylfurfural, decanoic acid ethyl ester, α-muurolene, acetic acid 2-phenylethyl ester, dodecanoic acid ethyl ester, and phenylethyl alcohol. Some of these compounds have also been found in mezcal samples from other agave species, for example, ethyl acetate and acetic acid were among the major compounds identified in *A. salmiana*, *A. angustifolia* Haw, and *A. potatorum*. Additionally, specific chemical substances, such as limonene, which was also found in this species, have been proposed as potential marker compounds [11,18,45].

Individually, 40 compounds were identified in the mezcal tobasiche (2021); 54 and 47 in the mezcals bicuishe 2017 and 2015, respectively, and 48 and 44 in the mezcals madrecuishe (2016) and (2018–2020), respectively. In the mezcal tobasiche, the compound 1,2,3-trimethyl-benzene was identified exclusively for this mezcal. In the mezcal bicuishe (2017), the compound nerolidol was found exclusively, which was not found in the mezcal from 2015, where the presence of linalool oxide was detected. These compounds may indicate the maturation time of the mezcal bicuishe. In the mezcal madrecuishe (2016), the presence of terpinolene was found exclusively, while in the madrecuishe (2018–2020), no compound was found that was produced exclusively in this mezcal. The magueyes mentioned above belong to the species *A. karwinskii* Zucc. In the mezcal espadín (2021) (*A. angustifolia* Haw), 48 compounds were identified, six of which were found only in this mezcal: isopropyl butyrate, γ-terpinene, acetic acid octyl ester, octanoic acid 3-methylbutyl ester, ethyl *trans*-4-decenoate, and 2-methylphenol. In the mezcal tepeztate (2021) (*A. marmorata* Roezl), 49 compounds were identified, two of which were produced exclusively in this mezcal: isobutyl acetate and 1,1-diethoxynonane. Finally, the mezcal tobalá (2015) (*A. potatorum*) produced 46 compounds, with the compound 4-methylpentanoic acid ethyl ester being exclusive to this mezcal. The compounds exclusively identified in certain mezcals, such as 1,2,3-trimethylbenzene, nerolidol, and terpinolene, act as specific chemical volatile compounds to differentiate mezcals based on the *Agave* species and maturation time. Although some of these compounds were detected in only one year, their presence is influenced by genetic, environmental, and technological factors [6,8,40], suggesting their potential as indicators of authenticity and maturation. However, further studies are required to evaluate their consistency across different harvests and production conditions.

The scientific literature indicates that mezcal production involves several critical stages that influence the chemical composition of the final product, particularly during distillation [8,43,44]. Regarding methanol content, all mezcals, except for bicuishe 2015 (Table 2), fall within the permissible limits established by the standard [32] (limits between 100 and 300 mg/100 mL of anhydrous alcohol). However, it has been proposed that this parameter should be modified, as the standard is based on non-distilled fermented beverages, such as wine, rather than distilled beverages [46]. Methanol due to their lower molecular weight and boiling point concentrate primarily in the head of the distillation, however, it is present throughout the distillation process and in tail fraction in significant quantities. Higher alcohols, esters, and aldehydes tend to appear in the initial fractions of the distillate, whereas furfural is predominant in the tail fraction [47,48]. In this regard, the furfural concentrations exceeding the maximum limit established by the standard [32] (maximum 5 mg/100 mL of anhydrous alcohol) in the analyzed mezcals from the species *A. karwinskii* Zucc. (Table 2) may indicate a late cut in the tail of the distillation, allowing these compounds to mix with the main distillate [49]. Proper control of the distillation process requires making precise cuts in the fractions to minimize the accumulation of furfural and methanol in the final product while preserving the sensory characteristics associated with higher alcohols and esters [8], this can be achieved by implementing an automatic temperature control mechanism in the distiller head. On the other hand, the concentrations of 1-propanol, 2-methyl-1-propanol, and 1-butanol (higher alcohols) are within the permissible limits of 100–500 mg/100 mL of anhydrous alcohol established by the standard [32]. It is important to note that, for these higher alcohols, the standard establishes a cumulative value for the total of higher alcohols. These results suggest proper management of the most important technological variables present in fermentation (temperature control, TSS monitoring, pH, and ethanol production) and the initial fractions of the distillate; however, they highlight the importance of improving the final cuts of distillation to fully comply with regulatory parameters and ensure product quality.

The quantification of VOCs, especially methanol, is essential to ensure the traceability and authenticity of mezcal, as adulteration with elevated methanol levels or unauthorized substances can have serious public health implications and undermine consumer trust. This study highlights the importance of applying GC-MS techniques, such as those used in this research, to identify specific chemical compounds related to the production process and agave species, distinguishing authentic products from imitations and reinforcing mezcal’s status as a beverage with a DO [2,6,8].

The main aroma descriptors reported in this study originate from different metabolic pathways, which are influenced by technological variables such as fermentation and distillation conditions [3,8,9,10,11] (Table 1 and Table S2). These compounds play a crucial role in defining the sensory profile of mezcal. Specifically, ethyl acetate, 1-butanol, acetic acid, and furfural exhibited the highest variability (*p* ≤ 0.05) among mezcals from different agave species and storage times (Table 2). This variation highlights the influence of both raw material composition and production processes on the final chemical profile. The presence and concentration of these key volatile compounds, as detailed in Table 3, directly impact the aroma and quality of mezcal, reinforcing the role of process control in maintaining product consistency and authenticity.

The magnitude of the variation is strongly influenced by the characteristics of the raw material used (e.g., agronomic management, physiological age, post-harvest handling), control of process variables (e.g., system pressure, temperature, time), and the handling conditions of the final product (e.g., temperature, time, type of packaging material, light exposure) [22,50]. On the other hand, methanol, diethyl acetal aldehyde, 1-propanol, and 2-methyl-1-propanol, although they did not show significant differences among the analyzed mezcals, act as robust indicators of consistency in production processes and as base descriptors of the product’s chemical profile. From a scientific perspective, this consistency suggests that they play a key role in preserving the quality and identity of the distilled spirit, as they reflect reproducible fermentation and distillation conditions. Their stability makes them valuable for quality control and authentication, ensuring the identity and homogeneity of the product [22].

Regarding physical properties, viscosity, total soluble solids, alcohol content, and density showed significant differences among the analyzed mezcals (Table 2). In terms of the relationship between volatile compounds and physical properties, previous research highlights that changes in viscosity and density may be related to the proportion of alcohols and esters, which contribute to the organoleptic balance and texture of mezcals [6,39].

**Figure 1:**
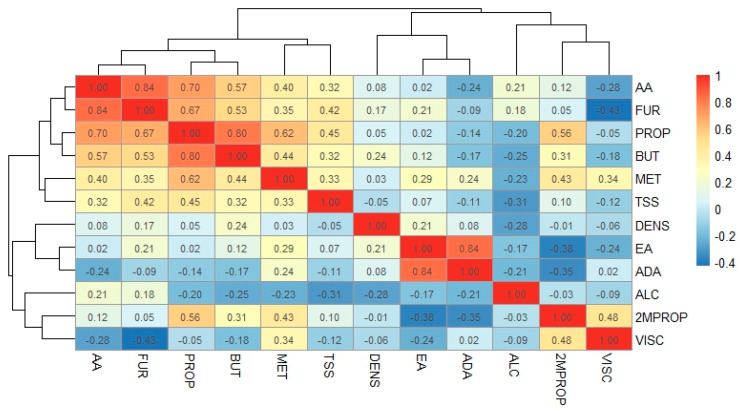
Correlation graph between physical properties and volatile compounds. AA: acetic acid, FUR: furfural, PROP: 1-propanol, BUT: 1-butanol, MET: methyl alcohol, TSS: total soluble solids, DENS: density, EA: ethyl acetate, ADA: acetaldehyde diethyl acetal, ALC: ethyl alcohol, 2MPROP: 2-methyl-1-propanol, and VISC: viscosity.

In Figure 1, the correlation graph (heatmap) of the physicochemical variables is shown. Two groups were observed: on one side, the concentrations of acetic acid (AA), furfural (FUR), 1-propanol (PROP), 1-butanol (BUT), methyl alcohol (MET), and total soluble solids (TSS) are clustered together; on the other side, the concentrations of ethyl acetate (EA), acetaldehyde diethyl acetal (ADA), ethyl alcohol (ALC), 2-methyl-1-propanol (2MPROP), the viscosity (VISC), and density (DENS) values of the mezcals form another group. A statistically significant correlation (*p* ≤ 0.5) is shown between AA and FUR (0.84) and EA and ADA (0.84), as well as the correlation between PROP and other variables such as BUT (0.80), AA (0.70), FUR (0.67), and MET (0.62). It is worth noting that the significant correlations are between volatile compounds, while those with physical properties are insignificant (less than 0.40).

According to the results obtained, EA was found in high concentrations in the tobalá samples, while in tobasiche and espadín, it was found in lower concentrations; the same behavior was observed in BUT, which was found at higher concentrations in mezcal samples with longer storage periods. EA and AA are among the majority compounds identified in *A. salmiana* [11,18,45]. Also, esters and aldehydes tend to appear in the initial fractions of the distillate [47,48]. PROP and BUT are compounds controlled by the standard and are found within permissible levels. The correlation between these compounds provides information on the identity of the mezcal samples studied and on all the factors related to the production of this artisanal product, such as the type of agave, fermentation management, distillation cuts to properly separate the heads, body, and tail, proper packaging of the final product, and the storage time of the mezcal. This information, along with the identified volatile compounds, is useful for creating a data system that serves as a starting point for identifying the mezcals produced in the study region.

Total soluble solids correlate positively with the concentration of PROP (0.45) and FUR (0.42). Density and alcohol content have a negative correlation, which is consistent with the recommendation of the international technical standard [51], as the decrease in mezcal density is on the order of 0.004 g/cm^3^ for every 1% increase in mezcal alcohol content. It is imperative to clarify that minor variations depend on the composition of the beverage. Interestingly, there is a negative and significant correlation between viscosity and furfural concentration (−0.43), and a positive correlation between viscosity and 2-methyl-1-propanol concentration (0.48), suggesting that this parameter may serve as an indicator of high furfural concentration in mezcals. The bicuishe 2016 mezcal, which had the highest furfural content, exhibited the lowest viscosity value (Table 2).

**Figure 2:**
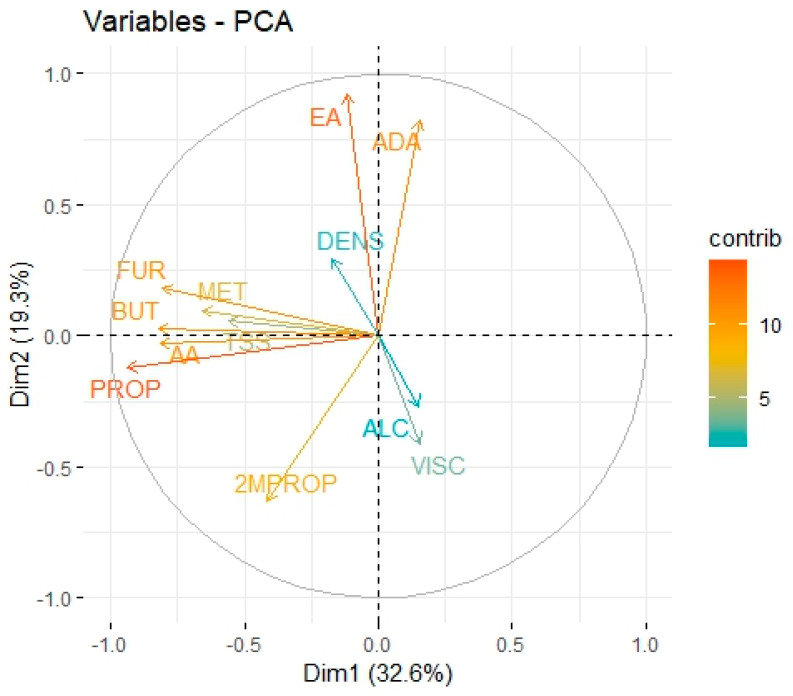
Principal component analysis. Variable correlation graph. EA: ethyl acetate, ADA: acetaldehyde diethyl acetal, MET: methyl alcohol, PROP: 1-propanol, 2MPROP: 2-methyl-1-propanol, BUT: 1-butanol, AA: acetic acid, and FUR: furfural, ALC: ethyl alcohol, VISC: viscosity, TSS: total soluble solids, and DENS: density.

Finally, physical properties, such as density and viscosity, which correlate with chemical composition, also contribute to the organoleptic profile of mezcal, emphasizing its authenticity. These findings reaffirm the importance of standardizing analytical methods and promoting traceability in production processes to ensure the quality of mezcal in competitive and high-value markets. Figure 2 shows the principal components plot of the main volatile compounds and physical variables analyzed. Dimension 1 explains 33% of the variability in the data set, with the most significant variables being: FUR, BUT, AA, and PROP (longer red and orange arrows); dimension 2 explains 19% of the variability, with the most significant variables being 2MPROP, EA, and ADA (longer red and orange arrows) (see Table S3 Supplementary Material). These two dimensions explain over 50% of the variability in the data set. The correlation map allows for the identification of bivariate relationships between the variables considered, that is, how volatile compounds and physical properties are associated with each other; however, it is limited to showing relationships between pairs of variables. PCA, for its part, provides global information on the data set; it simultaneously integrates all the variables and allows for the visualization of how the variables are grouped. This multivariate statistical tool allows for the detection of clustering or differentiation patterns among mezcal samples, and in turn identifies which variables have the greatest influence. The principal component analysis (PCA) shows that 1-propanol and ethyl acetate are the volatile compounds with the greatest contribution to mezcal aromas, while density and ethanol percentage have a minimal influence. 1-Propanol contributes alcoholic, solvent-like, fruity, and sweet aromatic notes to all mezcals, as no significant differences were observed among them (Table 2). On the other hand, ethyl acetate was found at higher levels in mezcal tobalá (*A. potatorum*), followed by bicuishe and madrecuishe (*A. karwinskii* Zucc.), imparting fruity flavors to the mezcals. This confirms that mezcals produced from these species exhibit fruity notes and elegant flavors [5,6,7].

**Figure 3:**
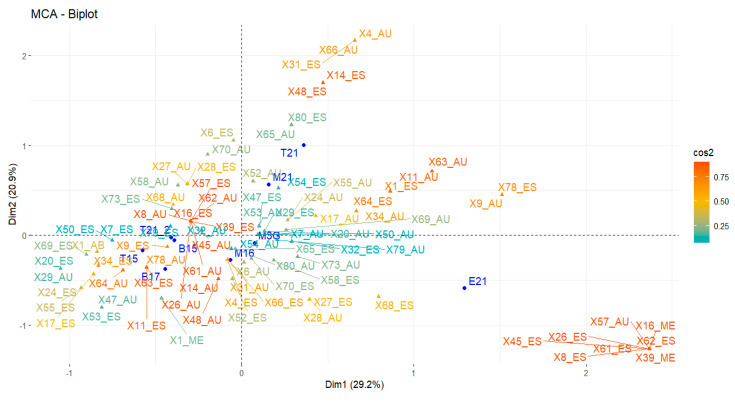
MCA analysis of qualitative data, where X represents volatile compounds (see Supplementary Material Table S2). Abundance of compounds: AU (Absent 0%), ES (Scarce ≤ 5%), ME (Medium 5–15%), and AB (Abundant > 15%). In dark blue, the agave species used for mezcal production are indicated: T15: tobalá (2015), B15: bicuishe (2015), B17: bicuishe (2017), M16: madrecuishe (2016), M3G: madrecuishe (2018-2020), M21: madrecuishe (2021), T21: tobasiche (2021), E21: espadín (2021), T21-2: tepeztate (2021).

Figure 3, based on a multiple correspondence analysis (MCA-Biplot), reveals clear associations between the identified volatile compounds and the species and subtypes of agave used in mezcal production. Additionally, a significant negative correlation was observed between viscosity and most of the evaluated chemical compounds, suggesting a complex interaction between physicochemical properties and aromatic profiles. While PCA enabled the identification of clustering patterns among mezcals based on quantitative variables (volatile compounds and physical properties), MCA provided a complementary perspective by exploring relationships between agave species and categorical presence levels (absent, medium, high) of key volatile compounds. MCA allowed for the identification of specific associations between particular species and the relative abundance of certain compounds, offering a more detailed qualitative characterization of each mezcal’s chemical profile. This qualitative approach is particularly valuable for understanding sensory variability and establishing links between chemical profiles and botanical origin, which are not accessible through PCA alone. Therefore, MCA complements PCA by offering a categorical perspective that enhances the traceability and differentiation of the mezcals analyzed. 

These results, consistent with previous research highlighting the influence of botanical origin and production process on mezcal’s chemical profiles [8], underscore the usefulness of multivariate tools for traceability and differentiation of spirits, as well as their role in ensuring product quality and authenticity.

The mezcal obtained from agave espadín 2021 (E21) (positive Dim1 values) is characterized by the presence of the compounds X45 (acetic acid ethyl ester), X26 (γ-terpinene), X8 (isopropyl butyrate), X61 (octanoic acid 3-methylbutyl ester), and X62 (ethyl *trans*-4-decenoate) in low concentrations (≤5%), X39 (octanoic acid ethyl ester) and X16 (3-methyl-1-butanol acetate) in medium concentrations (5–15%), and the absence of compound X57 (longifolene).

The tobasiche 2021 (T21) and madrecuishe 2021 (M21) mezcals are chemically similar (positive Dim1 values), both belonging to the species *A. karwinskii*. They share the compounds X1 (ethyl acetate), X15 (2-methyl-1-propanol), and X22 (2-methyl-1-butanol) in low percentages. The tepeztate 2021 (T21_2), bicuishe 2015 (B15), tobalá 2015 (T15), and bicuishe 2017 (B17) mezcals (negative Dim1 values) form another group characterized by the presence of the compounds X1 (ethyl acetate), X15 (2-methyl-1-propanol), X22 (2-methyl-1-butanol), X23 (3-methyl-1-butanol), X39 (octanoic acid ethyl ester), X41 (acetic acid), and X59 (decanoic acid ethyl ester) in high, medium, and low concentrations. Finally, the madrecuishe 2016 (M16) and madrecuishe 2018 (M3G) mezcals are grouped in the center and are characterized by the compounds X1 (ethyl acetate), X15 (2-methyl-1-propanol), X22 (2-methyl-1-butanol), X23 (3-methyl-1-butanol), and X41 (acetic acid) in low-abundance percentages.

With these results, we can confirm that mezcals produced from madrecuishe and tobasiche 2021 (*A. karwinskii* Zucc.) are chemically similar. The mezcal made from espadín 2021 (*A. angustifolia* Haw) is chemically different from the mezcals of the other species and subtypes. The mezcals tobalá 2015 (*A. potatorum*); Bicuishe 2015 and 2017 (*A. karwinskii* Zucc.), and tepeztate 2021 (*A. marmorata* Roezl.) are chemically similar; even though they are subtypes from different agave species. These mezcals are characterized by the presence of the compounds X1 (ethyl acetate); X15 (2-methyl-1-propanol); X22 (2-methyl-1-butanol); X23 (3-methyl-1-butanol); X39 (octanoic acid ethyl ester); X41 (acetic acid); and X59 (5-methylfurfural). The mezcal made from madrecuishe 2016 and madrecuishe 2018 (*A. karwinskii* Zucc.) are chemically different from the other mezcals

**Figure 4:**
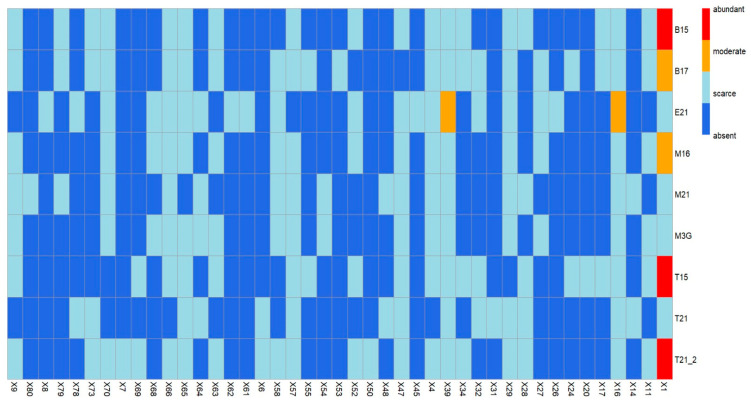
Heatmap of categorical variables and their distribution in agave species and subtypes. T15: tobalá (2015), B15: bicuishe (2015), B17: bicuishe (2017), M16: madrecuishe (2016), M3G: madrecuishe (2018, 19, 20), M21: madrecuishe (2021), T21: tobasiche (2021), E21: espadín (2021), T21-2: tepeztate (2021).

Finally, Figure 4 shows the heatmap of categorical variables for the volatile compounds that significantly contribute to the aroma and flavor of mezcals and their distribution across different species and subtypes of wild agave used. The compound X1 (ethyl acetate) is the most abundant in the mezcals from the species B15 (bicuishe 2015), T15 (tobalá 2015), and T21_2 (tepeztate 2021), and is present in moderate proportions in B17 (bicuishe 2017) and M16 (madrecuishe 2016). The compounds X39 (octanoic acid ethyl ester) and X16 (3-methyl-1-butanol acetate) are found in moderate proportions in E21 (espadín 2021). These results are consistent with those found in Figure 3, where the bicuishe, tobalá, tepeztate, and madrecuishe species share common VOCs, while the espadín species has VOCs that differentiate it from the others.

Viscosity becomes a distinctive parameter that can help predict the furfural and 2-methyl-propanol content in distilled spirits. However, to establish a correlation between these variables, the next step is to design experimental studies aimed at measuring both parameters within a controlled range of samples, considering factors such as temperature and compound interactions. These findings enable a detailed chemical characterization of agave species and can be used to associate the composition of mezcals with their respective raw materials [8].

## 4. Conclusions

This study provides a comprehensive characterization of the volatile profile and physicochemical properties of artisanal mezcal produced from four wild agave species in Oaxaca. A total of 82 volatile organic compounds were identified, with esters, higher alcohols, and terpenes being the most abundant. Among them, ethyl acetate, acetic acid, 1-butanol, and furfural exhibited significant variability (*p* ≤ 0.05) among different mezcal types, highlighting their potential as key volatile compounds for traceability and quality assessment. Additionally, compounds such as 1,2,3-trimethyl-benzene, nerolidol, and terpinolene were identified as unique indicators for distinguishing mezcal based on agave species and storage time. 

The results confirm that the volatile composition of mezcal is influenced by raw material, production process, and storage time. The correlation analysis between physicochemical properties and VOCs suggests that viscosity and density could serve as indirect indicators of the chemical complexity of mezcal. These findings underscore the importance of optimizing production parameters to maintain product authenticity and sensory consistency. Furthermore, the application of multivariate statistical tools allowed for a clear differentiation of mezcal samples, reinforcing the value of key chemical compounds in ensuring authenticity and compliance with regulatory standards. 

For the mezcal sector, the findings have direct practical implications. The identification of volatile compounds that characterize each agave species and their association with sensory properties paves the way for more precise quality control systems, more robust certification mechanisms, and strategies to enhance the product’s value in specialized markets. Furthermore, the knowledge generated can be leveraged by producers, regulators, and market stakeholders to safeguard mezcal’s authenticity, prevent adulteration, and promote sustainable production practices that highlight the biocultural richness of agave in Mexico.

## Figures and Tables

**Table 1:** Properties and origin of quantified volatile compounds in mezcals.

Name	Conferred Properties	References
Ethyl acetate	Ethyl acetate is produced by the yeast *Saccharomyces cerevisiae* through the enzyme acetyl alcohol transferase. It imparts fruity flavors and aromas to beverages.	[18,19,20]
Methanol	Agave contains pectin with methoxyl groups, which break down due to the high temperatures in ovens. Some yeasts have a pectin-methyl-esterase enzyme, which separates methoxyl groups from pectins and produces methanol during cooking.	[21]
Acetaldehyde diethyl acetal	Acetal is the primary compound produced during alcoholic fermentation. Its main biosynthesis occurs during the anabolic process through the enzyme pyruvate decarboxylase. Aldehydes are also produced during the maturation phase due to alcohol oxidation. Fruity aromatic note.	[18,21,22]
1-Propanol	It can be produced from amino acids through catabolism. Aromatic notes: alcoholic, solvent-like, fruit, and sweet. The production of higher alcohols such as propanol, isobutanol, and amyl alcohols is associated with ethanol production.	[22,23]
2-Methyl-1-propanol	Produced through amino acid catabolism, including aldehydes that are reduced by alcohol dehydrogenase into their respective alcohols, though at lower production rates than ethanol. Aromatic notes: sweet, chemical, bleach, chocolate, musty.	[24]
Acetic acid	Acetic acid originates from the Maillard reaction during cooking, as it has been identified in exudates of *Agave tequilana* Weber. Aromatic note: vinegar.	[18,25]
Furfural	Produced during thermal processes, occurring during both the cooking and distillation phases, due to the thermal degradation of sugars. Aromatic notes: floral, fruity.	[11,18]
1-Butanol	It can result from a combination of chemical processes, including aldehyde reduction to alcohols and micro-oxidation, along with a possible relative concentration effect due to the selective evaporation of volatile compounds caused by storage in a semi-permeable container. Aromatic note: sweet, fusel.	[18]

**Table 2:** Volatile compounds (mg/100 mL A.A.) and physical properties of artisanal mezcal from *A. potatorum*, *A. karwinskii* Zucc., *A. angustifolia* Haw and *A. marmorata* Roezl.

Mezcal Sample	Year of Production	EA	MET	ADA	PROP	2MPROP	BUT	AA	FUR	VISC (mPa∙s)	TSS (%)	ALC (% *v*/*v*)	DENS (g/cm^3^)
*A. potatorum*													
Tobalá	2015	503.54 ± 165.30 ^a^	257.81 ± 25.54 ^a^	41.35 ± 36.71 ^a^	32.1 ± 3.61 ^a^	89.3 ± 2.16 ^ab^	6.5 ± 1.86 ^a^	238.9 ± 26.30 ^abc^	4.9 ± 1.44 ^cd^	3.37 ± 0.02 ^ab^	15.93 ± 0.21 ^c^	44.83 ± 0.99 ^d^	0.93 ± 0.01 ^a^
*A. karwinskii* Zucc.													
Bicuishe	2015	263.16 ± 60.37 ^ab^	317.55 ± 183.12 ^a^	26.50 ± 11.95 ^a^	16.21 ± 9.92 ^a^	88.16 ± 33.39 ^ab^	1.99 ± 1.20 ^ab^	125.01 ± 26.64 ^bc^	2.40 ± 1.84 ^cd^	3.36 ± 0.01 ^ab^	15.97 ± 0.15 ^c^	45.93 ± 0.31 ^cd^	0.89 ± 0.02 ^ab^
	2017	320.80 ± 54.61 ^ab^	207.67 ± 7.97 ^a^	12.53 ± 2.16 ^a^	24.49 ± 1.98 ^a^	100.58 ± 9.85 ^a^	2.45 ± 0.60 ^ab^	266.13 ± 33.14 ^abc^	9.64 ± 2.10 ^abc^	3.41 ± 0.01 ^ab^	16.40 ± 0.0 ^a^	47.80 ± 0.20 ^b^	0.88 ± 0.03 ^b^
Madrecuishe	2016	338.70 ± 29.77 ^b^	239.97 ± 28.86 ^a^	20.69 ± 6.80 ^a^	27.64 ± 4.47 ^a^	71.39 ± 20.35 ^b^	2.05 ± 0.72 ^ab^	408.76 ± 82.88 ^a^	14.62 ± 2.95 ^a^	3.26 ± 0.29 ^b^	16.30 ± 0.10 ^ab^	47.23 ± 0.15 ^b^	0.92 ± 0.01 ^ab^
	2018–2020	372.77 ± 47.30 ^b^	175.06 ± 27.13 ^a^	19.80 ± 4.68 ^a^	31.77 ± 5.28 ^a^	102.15 ± 18.64 ^a^	3.57 ± 0.59 ^ab^	335.65 ± 29.73 ^ab^	13.28 ± 2.13 ^ab^	3.34 ± 0.25 ^ab^	16.23 ± 0.06 ^abc^	46.67 ± 0.21 ^bc^	0.92 ± 0.01 ^ab^
	2021	214.76 ± 16.92 ^b^	157.82 ± 12.46 ^a^	7.48 ± 1.92 ^a^	16.03 ± 4.31 ^a^	108.06 ± 12.90 ^a^	1.02 ± 0.37 ^b^	228.35 ± 30.51 ^abc^	5.57 ± 1.44 ^bcd^	3.38 ± 0.01 ^ab^	15.33 ± 0.06 ^d^	50.33 ± 0.99 ^a^	0.89 ± 0.03 ^ab^
Tobasiche	2021	178.96 ± 4.02 ^b^	124.82 ± 5.05 ^a^	20.22 ± 10.77 ^a^	23.52 ± 10.79 ^a^	141.97 ± 31.19 ^a^	0.00 ^b^	74.74 ± 9.31 ^c^	0.31 ± 0.14 ^cd^	3.37 ± 0.01 ^ab^	16.00 ± 0.01 ^bc^	45.43 ± 1.10 ^d^	0.91 ± 0.01 ^ab^
*A. angustifolia* Haw.													
Espadín	2021	190.78 ± 32.02 ^b^	211.94 ± 53.34 ^a^	28.15 ± 12.76 ^a^	23.55 ± 10.01 ^a^	127.98 ± 48.31 ^a^	1.02 ± 0.51^b^	142.66 ± 58.56 ^bc^	1.55 ± 0.48 ^cd^	3.62 ± 0.40 ^a^	15.97 ± 0.06 ^c^	45.67 ± 0.06 ^cd^	0.89 ± 0.04 ^ab^
*A. marmorata* Roezl.													
Tepeztate	2021	296.41 ± 31.35 ^ab^	120.43 ± 40.70 ^a^	30.44 ± 10.47 ^a^	23.02 ± 11.83 ^a^	113.24 ± 29.74 ^a^	1.06 ± 0.71 ^b^	174.47 ± 35.79 ^bc^	3.25 ± 1.35 ^cd^	3.37 ± 0.01 ^ab^	16.20 ± 0.40 ^abc^	47.83 ± 0.25 ^b^	0.90 ± 0.02 ^ab^

Mean ± SD. ^a, b, c, d^ Different superscripts within the same column indicate that the means differ significantly (*p* ≤ 0.05), A.A.:, anhydrous alcohol, EA: ethyl acetate, MET: methyl alcohol, ADA: acetaldehyde diethyl acetal, PROP: 1-propanol, 2MPROP: 2-methyl-1-propanol, BUT: 1-butanol; AA: acetic acid, FUR: furfural, VISC: viscosity, TSS: total soluble solids, ALC: ethyl alcohol, DENS: density.

**Table 3:** Concentration in mg/L of sample of the main volatile organic compounds (VOCs) in mezcals produced from different agave species and phenotypes.

Compound	Bicuishe 2017		Espadín 2021		Tobasiche 2021		Bicuishe 2015		Tepeztate 2021		Tobalá 2015		Madrecuishe 2021		Madrecuishe 2018–2020		Madrecuishe 2016	
	mg/L	mg/100 mL A.A.	mg/L	mg/100 mL A.A.	mg/L	mg/100 mL A.A.	mg/L	mg/100 mL A.A.	mg/L	mg/100 mL A.A.	mg/L	mg/100 mL A.A.	mg/L	mg/100 mL A.A.	mg/L	mg/100 mL A.A.	mg/L	mg/100 mL A.A.
Ethyl acetate	1681.45 ± 286.21	320.80 ± 54.61	1029.11 ± 172.71	190.78 ± 32.02	907.86 ± 20.40	178.96 ± 4.02	1425.79 ± 327.06	263.16 ± 60.37	1575.96 ±166.69	296.41 ± 31.35	2504.01 ± 821.99	503.54 ± 165.30	1166.87 ± 91.92	214.76 ± 16.92	1876.71 ± 238.11	372.77 ± 47.30	1758.02 ± 154.52	338.70 ± 29.77
Acetaldehyde diethyl acetal	65.70 ± 11.33	12.53 ± 2.16	151.86 ± 68.82	28.15 ± 12.76	102.60 ± 54.62	20.22 ± 10.77	143.56 ± 64.73	26.50 ± 11.95	161.84 ± 55.65	30.44 ± 10.47	205.61 ± 182.53	41.35 ± 36.71	40.63 ± 10.44	7.48 ± 1.92	99.70 ± 23.57	19.80 ± 4.68	107.38 ± 35.31	20.69 ± 6.80
Methyl alcohol	1088.51 ± 41.76	207.67 ± 7.97	1143.27 ± 287.72	211.94 ± 53.34	633.20 ± 25.63	124.82 ± 5.05	1720.49 ± 992.17	317.55 ± 183.12	853.76 ± 43.54	120.43 ± 40.70	1282.03 ± 127.03	257.81 ± 25.54	857.50 ± 67.67	157.82 ± 12.46	881.33 ± 136.57	175.06 ± 27.13	1245.58 ± 149.81	239.97 ± 28.86
1-propanol	128.34 ± 10.36	24.49 ± 1.98	127.05 ± 54.00	23.55 ± 10.01	119.34 ± 54.72	23.52 ± 10.79	87.80 ± 53.73	16.21 ± 9.92	163.16 ± 58.61	23.02 ± 11.83	212.99 ± 17.94	42.83 ± 3.61	87.08 ± 23.43	16.03 ± 4.31	159.92 ± 26.58	31.77 ± 5.28	143.46 ±23.23	27.64 ± 4.47
2-methyl-1-propanol	527.18 ± 51.62	100.58 ± 9.85	690.35 ± 260.60	127.98 ± 48.31	720.22 ± 158.24	141.97 ± 31.19	477.64 ± 180.92	88.16 ± 33.39	602.09 ± 158.14	113.24 ± 29.74	592.41 ± 10.74	119.13 ± 2.16	587.13 ± 70.12	108.06 ± 12.90	514.28 ± 93.84	102.15 ± 18.64	370.53 ± 105.64	71.39 ± 20.35
1-butanol	12.83 ± 3.14	2.45 ± 0.60	8.24 ± 0.41	1.02 ± 0.51	NQ	NQ	14.34 ± 6.62	1.99 ± 1.20	11.24 ± 3.40	1.06 ± 0.71	43.10 ± 9.26	8.67 ± 1.86	7.38 ± 0.97	1.02 ± 0.37	17.97 ± 2.97	3.57 ± 0.59	10.64 ± 3.75	2.05 ± 0.72
Acetic acid	1394.94 ± 173.72	266.13 ± 33.14	769.56 ± 315.87	142.66 ± 58.56	379.16 ± 47.22	74.74 ± 9.31	677.30 ± 144.34	125.01 ± 26.64	927.62 ± 190.31	174.47 ± 35.79	1584.45 ± 130.78	318.62 ± 26.30	1240.67 ± 165.77	228.35 ± 30.51	1689.85 ± 149.69	335.65 ± 29.73	2121.69 ± 430.20	408.76 ± 82.88
Furfural	50.54 ± 10.99	9.64 ± 2.10	8.38 ± 2.61	1.55 ± 0.48	2.08 ± 0.60	0.31 ± 0.14	12.98 ± 9.98	2.40 ± 1.84	17.30 ± 7.18	3.25 ± 1.35	32.61 ± 4.51	6.56 ± 0.91	30.28 ± 7.83	5.57 ± 1.44	66.88 ± 10.74	13.28 ± 2.13	75.86 ± 15.31	14.62 ± 2.95

Mean ± SD, A.A.: anhydrous alcohol, NQ: not quantifiable.

## Data Availability

The original contributions presented in the study are included in the article/Supplementary Material, further inquiries can be directed to the corresponding authors.

## Supplementary Materials

The following supporting information can be downloaded at https://www.mdpi.com/article/10.3390/foods14071222/s1. Table S1. Calibration information for target compounds. Table S2. Major compounds in mezcals derived from wild agave species. Table S3. Loading values of principal component analysis (PCA), eigenvalues, and percentage variability for the three main components (PC1-PC3) for physicochemical variables.
