# Supplementary material for: Chemistry of Mezcal: Volatile Profile of Artisanal Mezcal Made from Wild Agaves of Oaxaca
Source: Foods. 2025 Mar 31;14(7):1222. doi: 10.3390/foods14071222 (PMC11988476; doi:10.3390/foods14071222)
Supplement: Supplementary file 1 [file foods-14-01222-s001.zip › foods-3536605-supplementary.pdf]

Supporting Information

Chemistry of Mezcal: Volatile Profile of Artisanal Mezcal Made from Wild Agaves of Oaxaca

Rosa Elvira Sánchez-Fernández [1,*], Artemio Pérez-López [2,*], Anabel Morales-Solis [2], Yesenia Manilla-Tellez [2], Erika Daniela Reyes-Carmona [2] and Graciela Avila-Uribe [3]

[1] Laboratorio Nacional de Investigación y Servicio Agroalimentario y Forestal (LANISAF), Universidad Autónoma Chapingo, km 38.5, Carretera Mexico-Texcoco, Texcoco 56230, Mexico
[2] Posgrado en Ciencia y Tecnología Agroalimentaria, Departamento de Ingeniería Agroindustrial, Universidad Autónoma Chapingo, km 38.5, Carretera Mexico-Texcoco, Texcoco 56230, Mexico
[3] Estancia Posdoctoral Por Mexico, Secretaría de Ciencia, Humanidades, Tecnología e Innovación, Posgrado en Ciencia y Tecnología Agroalimentaria, Universidad Autónoma Chapingo, km 38.5, Carretera Mexico-Texcoco, Texcoco 56230, Mexico
* Correspondence: resf2012@gmail.com (R.E.S.-F.); aperezl.dia@gmail.com (A.P.-L.)

Table S1. Calibration information for target compounds.

Name	RT (min)	R^2	Equation
Ethyl acetate	7.06	0.9975	y = 17289x + 650958
Acetaldehyde diethyl acetal	7.17	0.9991	y = 65200x + 1000000
Methyl alcohol	7.39	0.9994	$y = 0.2248x^2 + 601.79x - 14339$
1-propanol	11.25	0.9973	y = 5740.3x - 388236
2-methyl-1-propanol	13.01	0.9945	y = 11449x - 280618
1-butanol	14.53	0.9946	y = 8752.2x - 46175
Acetic acid	21.71	0.9911	y = 5506.7x - 175504
Furfural	22.04	0.9936	y = 34692x + 282014

RT: retention time. R^2: correlation coefficient.

Table S2. Major compounds in mezcals derived from wild agave species.

							A. karwinskii						*A. angustifolia*	*A. marmorata*	*A. potatorum*
Com-pound (X)	Name	Formula	MW	KRI	KRI L	Ident	Tobasiche (2021)	Bicuishe (2017)	Bicuishe (2015)	Madrecui-she (2016)	Madrecuish e (2018-2020)	Madrecui-she (2021)	Espadín (2021)	Tepeztate (2021)	Tobalá (2015)
1	Ethyl Acetate	$C_4H_8O_2$	88.1	882.2	880	STD	1.24	6.00	5.72	9.40	4.86	2.09	4.60	4.23	20.84
2	Acetaldehyde diethyl acetal	$C_6H_{14}O_2$	118.1	895.2	889	STD	1.42	1.01	6.62	2.85	1.15	0.45	3.75	0.72	1.25
3	Methyl Alcohol	CH_4O	32.0	906.9	905	STD	0.16	0.64	1.83	0.59	0.26	0.37	0.45	0.50	2.95
4	2-methyl-Propanoic acid ethyl ester	$C_6H_{12}O_2$	116.1	978.5	976	Tentative	-	0.97	0.83	1.04	0.23	0.06	0.09	0.22	0.73
5	1,1-Diethoxy-2-methylpropane	$C_8H_{18}O_2$	146.1	985.7	976	Tentative	-	0.10	0.04	-	-	0.10	0.14	-	-
6	1,1-Diethoxybutane	$C_8H_{18}O_2$	146.1	985.6	988	Tentative	0.09	-	-	-	-	-	-	0.08	-
7	Isobutyl acetate	$C_6H_{12}O_2$	116.1	1018.6	1013	Tentative	-	-	-	-	-	-	-	0.10	-
8	Isopropyl butyrate	$C_7H_{14}O_2$	130.1	1044.3	1039	Tentative	-	-	-	-	-	-	0.03	-	-
9	Butanoic acid ethyl ester	$C_6H_{12}O_2$	116.1	1044.9	1044	Tentative	-	1.16	1.11	0.64	0.33	0.08	-	0.18	4.06
10	1-Propanol	C_3H_8O	60.1	1029.2	1049	STD	0.16	0.11	0.35	0.21	0.13	0.11	0.19	0.58	0.86
11	2-methylbutanoic acid ethyl ester	$C_7H_{14}O_2$	130.1	1060.5	1062	Tentative	-	0.17	0.13	0.16	0.09	-	-	0.02	0.12
12	3-methylbutanoic acid ethyl ester	$C_7H_{14}O_2$	130.1	1075.4	1076	Tentative	-	0.76	0.39	0.54	0.19	0.05	-	-	0.29
13	1,1-Diethoxy-3-methylbutane	$C_9H_{20}O_2$	160.1	1082.1	1074	Tentative	0.17	0.30	0.17	0.27	0.14	0.15	0.23	0.18	-
14	2,2,5-trimethyl-3,4-Hexanedione	$C_9H_{16}O_2$	156.1	1091.7	1100	Tentative	0.08	-	-	-	-	0.07	-	-	-

15	2-methyl-1-Propanol	$C_4H_{10}O$	74.1	1105.8	1114	STD	1.75	1.58	2.17	1.30	0.94	1.32	2.41	2.66	3.04
16	3-methyl-1-Butanol acetate	$C_7H_{14}O_2$	130.1	1124.6	1122	STD	0.90	2.42	3.66	1.03	1.06	0.92	12.15	3.14	0.72
17	Pentanoic acid ethyl ester	$C_7H_{14}O_2$	130.1	1138.0	1142	Tentative	-	0.09	0.10	-	-	-	-	-	0.84
18	4-Carene	$C_{10}H_{16}$	136.1	1147.5	1149	Tentative	0.74	1.02	0.73	0.91	0.38	0.91	0.22	0.14	0.04
19	1-butanol	$C_4H_{10}O$	74.1	1158.4	1150	STD	0.02	0.02	0.03	0.02	0.02	0.01	0.01	0.87	0.25
20	4-methylpentanoic acid ethyl ester	$C_8H_{16}O_2$	144.1	1191.9	1180	Tentative	-	-	-	-	-	-	-	-	0.26
21	Limonene	$C_{10}H_{16}$	136.1	1193.5	1199	Tentative	0.12	6.32	0.88	6.74	1.17	0.35	0.68	0.37	0.24
22	2-methyl-1-Butanol	$C_5H_{12}O$	88.1	1220.9	1218	STD	2.70	2.32	2.03	1.82	1.00	1.18	3.49	2.24	1.25
23	3-methyl-1-Butanol	$C_5H_{12}O$	88.1	1220.6	1218	STD	7.46	5.64	10.46	7.95	3.36	4.59	5.39	4.18	5.68
24	1,1-Diethoxyhexane	$C_{10}H_{22}O_2$	174.2	1237.9	1235	Tentative	-	0.11	-	-	-	-	-	-	0.07
25	Hexanoic acid ethyl ester	$C_8H_{16}O_2$	144.1	1237.3	1241	Tentative	0.10	0.87	0.93	0.80	0.42	0.12	0.45	0.23	1.09
26	γ-Terpinene	$C_{10}H_{16}$	136.1	1245.0	1238	Tentative	-		-			-	0.34	-	-
27	Bicyclo[4.2.0]octa-1,3,5-triene	C_8H_8	104.1	1261.8	1269	Tentative	-	1.00	-	0.89	0.66	-	2.64	-	-
28	Phenylbutanedioic acid	$C_{10}H_{10}O_4$	194.1	1263.9	1269	Tentative	0.60		0.07	-	-	0.54	-	0.41	0.12
29	*p*-Cymene	$C_{10}H_{14}$	134.1	1273.9	1272	Tentative	0.24	0.21	0.10	0.22	0.11	0.08	0.63	0.09	-
30	Isoterpinolene	$C_{10}H_{16}$	136.1	1285.6	1286	Tentative	-	-	0.10	-	-	0.14	0.24	-	-
31	1,2,3-Trimethylbenzene	C_9H_{12}	120.1	1285.0	1285	Tentative	0.09	-	-	-	-	-	-	-	-
32	1,2,4-Trimethylbenzene	C_9H_{12}	120.1	1285.6	1285	Tentative	0.09	0.06	-	-	-	-	0.06	-	0.08

33	Terpinolene	$C_{10}H_{16}$	136.1	1285.7	1280	Tentative	-	-	-	0.19	-	-	-	-	-
34	1,1,3-Triethoxypropane	$C_9H_{20}O_3$	176.1	1311.1	1297	Tentative	-	0.06	0.07	-	-	-	-	0.34	0.10
35	Heptanoic acid ethyl ester	$C_9H_{18}O_2$	158.1	1338.4	1332	Tentative	0.09	0.33	-	0.34	0.18	0.07	0.08	0.11	0.43
36	Ethyl lactate	$C_5H_{10}O_3$	118.1	1350.1	1352	Tentative	-	1.57	4.50	0.45	0.49	0.66	-	-	0.51
37	1-Hexanol	$C_6H_{14}O$	102.1	1363.5	1355	STD	-	-	-	0.16	0.07	-	-	-	-
38	3-methyl-1-Pentanol	$C_6H_{14}O$	102.1	1364.3	1348	Tentative	-	-	-	0.19	0.12	-	-	-	-
39	Octanoic acid ethyl ester	$C_{10}H_{20}O_2$	172.1	1439.8	1441	Tentative	1.83	2.61	3.60	4.18	2.50	0.92	7.59	1.00	1.01
40	*p*-Cymenene	$C_{10}H_{12}$	132.1	1446.2	1455	Tentative	-	0.12	0.08				0.26		
41	Acetic acid	$C_2H_4O_2$	60.0	1455.8	1449	STD	0.60	2.01	5.09	3.66	1.47	2.06	1.35	1.56	1.99
42	Linalool oxide	$C_{10}H_{18}O_2$	170.1	1460.5	1478	Tentative	-	-	0.44	-	-	-	-	-	-
43	2-Furaldehyde diethyl acetal	$C_9H_{14}O_3$	170.1	1469.8	1453	Tentative	-	0.05	-	0.65	-	-	-	0.01	0.05
44	Furfural	$C_5H_4O_2$	96.0	1474.3	1480	STD	0.01	0.32	0.16	0.76	0.31	0.13	0.12	0.09	0.22
45	Acetic acid octyl ester	$C_{10}H_{20}O_2$	172.1	1480.8	1476	Tentative	-	-	-	-	-	-	0.15	-	-
46	Ylangene	$C_{15}H_{24}$	204.2	1484.5	1488	Tentative	0.07	-	-	0.65	-	-	-		
47	Cyclosativene	$C_{15}H_{24}$	204.2	1498.9	1491	Tentative	8.37	-	-	2.04	0.65	0.69	0.91	0.49	0.10
48	Copaene	$C_{15}H_{24}$	204.2	1506.5	1501	Tentative	0.43	-	-	-	-	0.32	-	-	-
49	1-(2-furanyl)-Ethanone	$C_8H_{10}O_2$	110.0	1522.0	1512	Tentative	-	0.25	-	0.26	-	-	-	0.07	0.14
50	1,1-Diethoxynonane	$C_{13}H_{28}O_2$	216.2	1530.0	1514	Tentative	-	-	-	-	-	-	-	0.02	
51	Nonanoic acid ethyl ester	$C_{11}H_{22}O_2$	186.2	1543.6	1541	Tentative	0.67	1.11	0.86	1.20	0.55	0.21	0.49	0.31	0.80

52	Linalool	$C_{10}H_{18}O$	154.1	1552.0	1554	STD	-	-	0.86	0.25	-	-	0.39	0.40	0.33
53	Nerolidol	$C_{15}H_{26}O$	222.2	1552.6	1554	Tentative	-	0.79	-	-	-	-	-	-	-
54	2-hydroxy-4-methyl-Pentanoic acid ethyl ester	$C_8H_{16}O_3$	160.1	1555.8	1547	Tentative	-	-	-	-	0.07	0.17	-	-	-
55	Propionic acid	$C_3H_6O_2$	74.1	1540.0	1540	STD	-	0.10	-	-	-	-	-	-	0.24
56	5-methylfurfural	$C_6H_6O_2$	110.0	1590.6	1597	Tentative	0.22	0.50	0.38	1.27	0.04	0.22	0.23	0.10	0.19
57	Longifolene	$C_{15}H_{24}$	204.2	1594.5	1590	Tentative	0.45	0.52	0.47	0.74	0.60	0.58	-	0.29	0.35
58	β-Elemene	$C_{15}H_{24}$	204.2	1605.1	1600	Tentative	-	0.11	-	0.18	0.10	0.12	0.14	0.09	-
59	Decanoic acid ethyl ester	$C_{12}H_{24}O_2$	200.2	1647.1	1643	Tentative	1.27	4.40	1.65	3.91	1.62	0.52	6.44	1.04	1.98
60	1-Nonanol	$C_9H_{20}O$	144.2	1667.8	1666	Tentative	-	0.11	-	0.16	-	-		-	-
61	Octanoic acid 3-methylbutyl ester	$C_{13}H_{26}O_2$	214.2	1669.7	1671	Tentative	-	-	-	-	-	-	0.16	-	-
62	Ethyl *trans*-4-decenoate	$C_{12}H_{22}O_2$	198.2	1675.7	1699	Tentative	-	-	-	-	-	-	0.19	-	-
63	Estragole	$C_{10}H_{12}O$	148.1	1688.0	1685	Tentative	-	0.22	0.11	0.36	0.21	-	-	0.02	0.17
64	β-Acoradiene	$C_{15}H_{24}$	204.2	1692.5	1689	Tentative	1.25	-	-	-	0.09	0.37	0.29	-	-
65	γ-Muurolene	$C_{15}H_{24}$	204.2	1712.2	1715	Tentative	0.51	0.12	0.05	0.24	0.12	-	0.23	0.07	0.06
66	α-Terpineol	$C_{10}H_{18}O$	154.1	1716.7	1710	Tentative	-	0.32	0.41	0.37	0.14	0.06	0.52	0.24	0.15
67	α-Muurolene	$C_{15}H_{24}$	204.2	1746.9	1751	Tentative	0.48	0.15	0.05	0.16	0.16	0.20	0.27	0.07	0.09
68	Eremophilene	$C_{15}H_{24}$	204.2	1753.3	1751	Tentative	-	-	-	0.15	0.16	-	0.24	-	-
69	α-Curcumene	$C_{15}H_{22}$	202.2	1790.7	1798	Tentative	-	-	-	-	-	-	-	0.02	0.04
70	Methyl salicylate	$C_8H_8O_3$	152.0	1811.2	1804	STD	-	0.37	0.13	0.35	0.18	0.08	0.33	0.05	-

71	Acetic acid 2-phenylethyl ester	$C_{10}H_{12}O_2$	164.1	1842.3	1835	Tentative	0.18	0.93	0.70	0.85	0.46	0.17	2.44	2.27	0.20
72	Dodecanoic acid ethyl ester	$C_{14}H_{28}O_2$	228.2	1851.8	1850	Tentative	0.35	0.29	0.15	0.41	0.17	0.10	0.64	0.13	0.28
73	Calamenene	$C_{15}H_{22}$	202.2	1863.8	1853	Tentative	0.06	0.02	0.01	-	-	-	-	0.01	-
74	1/2-methyl-Naphthalene	$C_{11}H_{10}$	142.1	1895.9	1893	Tentative	0.75	0.16	0.08	0.17	0.14	0.50	1.42	0.09	0.22
75	Benzenepropanoic acid ethyl ester	$C_{11}H_{14}O_2$	178.1	1914.5	1914	Tentative	-	0.02	0.01	-	-	-	-	-	-
76	Phenylethyl Alcohol	$C_8H_{10}O$	122.1	1941.4	1944	Tentative	0.36	0.71	0.51	1.01	0.30	0.24	0.70	0.24	0.35
77	β-Calacorene	$C_{15}H_{20}$	200.2	1952.7	1954	Tentative	0.04	-	0.01	-	-	-	0.02	0.01	-
78	2,3-Dimethylnaphthalene	$C_{12}H_{12}$	156.1	2008.7	2008	Tentative	0.05	-	-	-	-	-	0.20	-	-
79	1,6-Dimethylnaphthalene	$C_{12}H_{12}$	156.1	2009.7	2006	Tentative	-	0.01	-	-	-	0.04	-	-	-
80	2-Methylphenol	C_7H_8O	108.1	2017.3	2014	Tentative	-	-	-	-	-	-	0.06	-	-
81	1,4-Dimethylnaphthalene	$C_{12}H_{12}$	156.1	2043.7	2041	Tentative	-	0.004	-	-	-	-	-	0.004	0.11
82	2,6-Dimethylnaphthalene	$C_{12}H_{12}$	156.1	2048.8	2038	Tentative	-	0.02	0.01	-	-	-	-	0.01	-

MW: Molecular weight in g/mol, KRI: Experimental Kovats retention index, KRIL: Kovats retention index reported in the literature. STD: identification was performed using standards.

Table S3. Loading values of Principal Component Analysis (PCA), eigenvalues, and percentage variability for the three main components (PC1-PC3) for physicochemical variables.

**Variable**	**PC1**	**PC2**	**PC3**
EA	-0.060	0.604	0.111
ADA	0.077	0.543	0.265
MET	-0.332	0.064	0.394
PROP	-0.476	-0.080	0.089
2MPROP	-0.210	-0.414	0.337
BUT	-0.416	0.016	0.025
AA	-0.412	-0.019	-0.284
FUR	-0.408	0.119	-0.310
ALC	0.077	-0.177	-0.411
TSS	-0.283	0.038	0.026
DENS	-0.088	0.194	0.070
VISC	0.078	-0.270	0.537
Eigenvalues	15.647	10.545	9.8963
Variability %	24.448	16.476	15.463

AA: acetic acid, FUR: furfural, PROP: 1-propanol, BUT: 1-butanol, MET: methyl alcohol, TSS: total soluble solids, DENS: density, EA: ethyl acetate, ADA: acetaldehyde diethyl acetal, ALC: ethyl alcohol, 2MPROP: 2-methyl-1-propanol, and VISC: viscosity.
